# Account of Diseases in an Eastern District of London, from the 20th of February to the 20th of March, 1801

**Published:** 1801-04

**Authors:** 


*Account of Diſeaſes in an Eaſtern Diſtrict of London, from the 20th of February to the 20th of March,* 1801.

| | No. of Caſes. | | No. of Caſes. |
|---|---|---|---|
| ACUTE DISEASES. | | Hyſteria - - - - - - - | 3 |
| Typhus - - - - - - | 20 | Apoplexia - - - - - - | 1 |
| Varicella - - - - - - | 1 | Fluor Albus - - - - - | 7 |
| Pneumonia - - - - - - | 6 | Amenorrhœa - - - - - | 6 |
| Cynanche Tonſillaris - - | 1 | Menorrhagia - - - - - | 2 |
| Inteſtinal Hæmorrhagy - | 1 | Prolapſus Vaginæ - - - - | 1 |
| CHRONIC DISEASES. | | Sciatica - - - - - - - | 2 |
| Cough - - - - - - - | 25 | Rheumatiſmus - - - - | 20 |
| Dyſpnœa - - - - - - | 14 | PUERPERAL DISEASES. | |
| Cough and Dyſpnœa - | 17 | Ephemera - - - - - - | 4 |
| Pleurodyne - - - - - - | 4 | Menorrhagia Lochialis - | 3 |
| Phthiſis Pulmonalis - - | 5 | Lochiorum Suppreſſio - - | 1 |
| Hydrothorax - - - - - | 3 | Mania - - - - - - - | 1 |
| Aſcites - - - - - - - | 2 | Rhagas Papillæ - - - - | 4 |
| Anaſarca - - - - - - | 2 | INFANTILE DISEASES. | |
| Hepatitis Chronica - - | 1 | Ophthalmia - - - - - - | 3 |
| Diarrhœa - - - - - - | 12 | Ophthalmia Purulenta - - | 2 |
| Cephalæa - - - - - - | 4 | Aphthæ - - - - - - - | 3 |

We had occaſion to remark in our laſt Report, that the change which had taken place in the temperature of the atmoſphere, would probably increaſe the number, and aggravate the ſymptoms of thoſe diſeaſes which generally prevail at this ſeaſon of the year. What it was but natural to expect, has actually taken place. Thoſe perſons, who from the mildneſs of the ſeaſon entertained the hope of paſſing through the winter without a recurrence of thoſe diſorders to which they had heretofore been liable, were reminded of their conſtitutional infirmity upon the approach of the froſt.

Catarrhal affections, Peripneumonies, Phthiſis Pulmonalis, and other morbid affections of the organs of reſpiration have increaſed in their number, or have been attended with an aggravation of

ſymptoms.

ſymptoms. The froſt, however, which was ſufficient to introduce this claſs of diſeaſes, was too ſhort in its continuance to produce a favourable change in the ſtate of another claſs. A fever, which has long been conſidered as the prevailing epidemic, ſtill prevails, and thoſe alarming affections of the head, which we have had ſuch frequent occaſion to remark, ſtill continue to form a diſtinguiſhing characteriſtic of the diſeaſe.

Varicella or Chicken Pox, a ſingle caſe of which appears in our liſt, is a diſeaſe of ſo mild a nature as not often to require much medical attention. It is generally introduced by a ſlight degree of fever, attended with trifling pain in the head or back, ſometimes with ſlight cough and a general laſſitude. About the third day an eruption appears on ſome part of the body, which in its progreſs aſſumes rather the appearance of veſicles than puſtules, which arrive very ſoon at the ſtate of maturation, and diſappear more haſtily than the ſmall-pox. In the inſtance referred to in the liſt, this diſeaſe, which occurred to a child of nine years old, aſſumed a more unpleaſant appearance. A conſiderable degree of fever preceded the eruption, and this was of ſuch a nature as not to be eaſily diſtinguiſhed at firſt from the Small-pox. The number of puſtules, together with their ſize and appearance, might, for a time, have led to a doubt reſpecting the nature of the diſeaſe; there was ſufficient evidence, however, of this patient having been before infected by the Small-pox.

From ſuch puſtules, very probably, matter has been ſometimes taken, with a view to inoculate the Small-pox; and the patient, ſome time after receiving this ſlighter diſeaſe, has been affected by the Small-pox, and an opinion has been entertained that, in this inſtance, the infection has been received a ſecond time.

